# Role of Dual Energy Computed Tomography Imaging in the Diagnosis of Gout

**DOI:** 10.7759/cureus.985

**Published:** 2017-01-20

**Authors:** Divya Jayakumar, Shiv T Sehra, Suneesh Anand, Gary W. Stallings, Abhijeet Danve

**Affiliations:** 1 Westchester Medical Center, New York Medical College; 2 Mount Auburn Hospital, Harvard Medical School; 3 Covenant Medical Center, Central Michigan University; 4 New York Medical College; 5 Yale New Haven Hospital, Yale University School of Medicine

**Keywords:** gout, monosodium urate crystals, dual energy ct, dect

## Abstract

Gout is a well-known inflammatory arthritis and affects four percent of the United States population. It results from the deposition of uric acid crystals in joints, tendons, bursae, and other surrounding tissues. Prevalence of gout has increased in the recent decade. Gout is usually seen in conjunction with other chronic comorbid conditions like cardiac disease, metabolic syndrome, and renal disease. The diagnosis of this inflammatory arthritis is confirmed by visualization of monosodium urate (MSU) crystals in the synovial fluid. Though synovial fluid aspiration is the standard of care, it is often deferred because of inaccessibility of small joints, patient assessment during intercritical period, or procedural inexperience in a primary care office. Dual energy computed tomography (DECT) is a relatively new imaging modality which shows great promise in the diagnosis of gout. It is a good noninvasive alternative to synovial fluid aspiration. DECT is increasingly useful in diagnosing cases of gout where synovial fluid fails to demonstrate monosodium urate crystals. In this article, we will review the mechanism, types, advantages, and disadvantages of DECT.

## Introduction and background

Gout is a crystal-induced inflammatory arthritis, which is one of the oldest and commonest rheumatic diseases [1-2]. The prevalence of gout is steadily increasing and it currently affects approximately 8.3 million individuals (3.9%) in the United States [2]. Deposition of monosodium urate (MSU) crystals in the joint spaces and tissues is the hallmark of gout. Gout is characterized clinically by acute onset of inflammatory mono-/ oligoarthritis and is often difficult to distinguish from other inflammatory arthritides without confirmation by synovial fluid analysis. Synovial fluid aspiration and visualization of MSU crystals has long been recognized as the gold standard for gout diagnosis. There are limited options to confirm the suspicion of gout when patients present during the intercritical phase. Asymptomatic hyperuricemia is common and only a few percent of those patients progress to develop gout. Recent advances in technology have led us to the use of energy rays to specifically detect MSU crystals in tendons, joints, bursae, and soft tissues. Dual energy computed tomography (DECT) is an upcoming imaging modality in rheumatology and shows great promise in diagnosing challenging cases of gout [3-5]. In this review, we will discuss the mechanism, types, advantages, and disadvantages of DECT.

## Review

**Figure 1:**
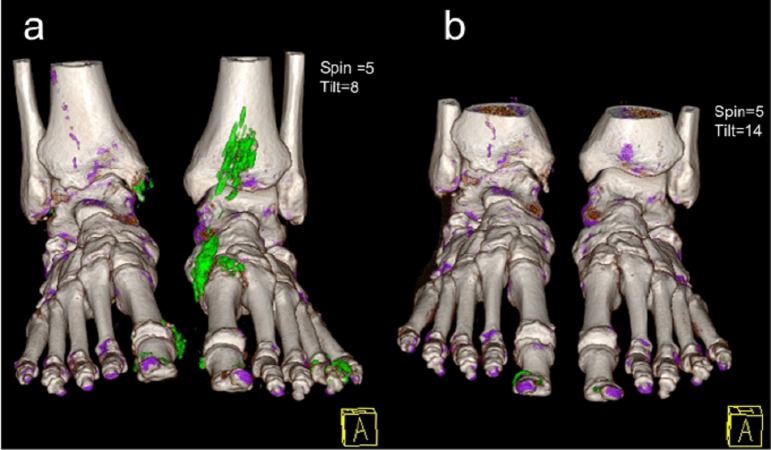
3D DECT reconstruction images Image showing a patient with tophaceous gout previous to (a) and 16 months after treatment with febuxostat (b). Image 1b shows complete resolution of the tophi (areas of green pixels around the nails represent typical artifacts); Purple: calcium; Green: MSU deposits; A: anterior (Reproduced with permission from journal-Gout and Hyperuricemia).

DECT is a modified computed tomography (CT) scan that utilizes two X-rays instead of one, as seen in a standard CT scan. It uses a regular X-ray and a lower energy X-ray at 140 kV and 80 kV to produce images of different types of tissues [6-7]. The difference in photoelectric absorption between calcium and urate allows measurable attenuation differences between urate and bone [8]. An image is thus created by distinctively separating and color coding calcium from monosodium urate. A DECT scan is considered positive by the presence of color-coded monosodium urate at joints and periarticular spaces [9]. In Figure 1, the MSU crystals are demonstrated in green while the calcium is demonstrated in purple. The color enhanced positivity reflects dense packaging of MSU crystals by extracellular neutrophil traps (NETs) [10]. DECT protocol is usually limited to the affected joint area. Comprehensive scanning for research purposes can include bilateral hands and wrists, elbows, knees, feet, and ankles [11-12]. This helps detect MSU deposits in multiple articular and periarticular sites by a single scan [13]. A DECT scan of joints like the spine and shoulders are rarely clinically indicated and therefore are not studied extensively.

DECT is an innovative imaging technique with a reported sensitivity of 84%–90% and specificity of 83%–93% [14-15]. It has recently made its way to the 2015 American College of Rheumatology (ACR)/European League against Rheumatism (EULAR) diagnostic criteria for gout. The target population fulfilling the diagnostic criteria are those patients demonstrating at least one episode of swelling, pain or tenderness in a bursa or joint space [9]. The detection of MSU crystals in the symptomatic joints on DECT scan is sufficient for the diagnosis of gout [9]. This is based on the Study for Updated Gout Classification Criteria (SUGAR) and includes clinical, laboratory, and imaging characteristics of gout. The new ACR/EULAR classification criteria showed a sensitivity of 92% and specificity of 89% [9, 16]. A study by Petsch, et al. observed that a considerable number of seronegative rheumatoid arthritis (RA) patients with hyperuricemia had periarticular MSU crystal deposits in DECT scan. This finding suggested that some patients fulfilling ACR/EULAR criteria for RA may be suffering from polyarticular gout rather than RA [13].

**Figure 2:**
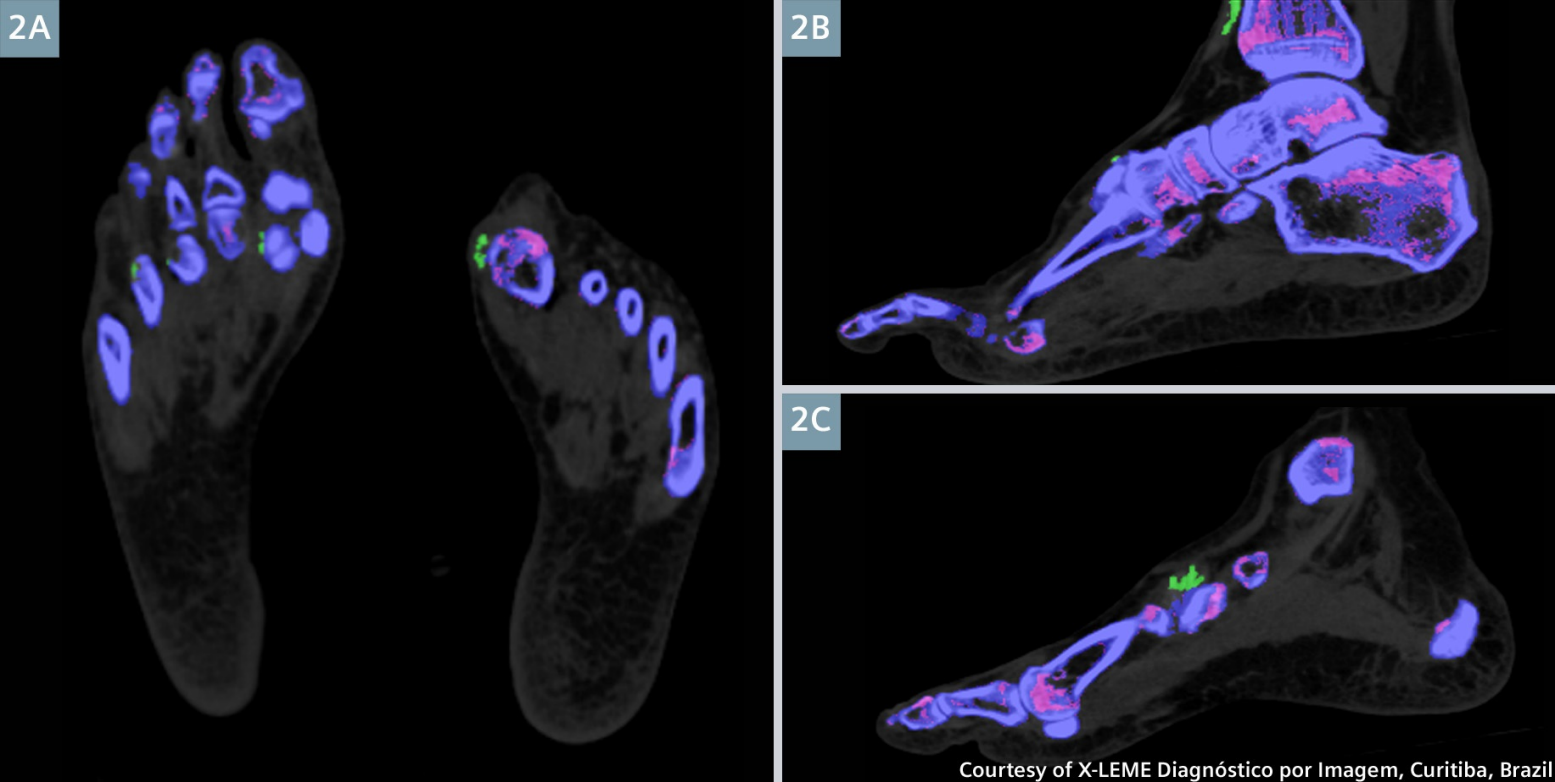
DECT multiplanar reformation (MPR) Images revealing multiple MSU tophi formations (in green) in the first left metatarsophalangeal joint (2a), in front of the right tibia (2b) and around the right medial cuneiform (2c). Smaller lesions were also seen in the right 1st and 4th metatarsophalangeal joints (2a). (Reproduced with permission from SOMATOM Sessions and X-LEME Diagnóstico por Imagem, Curitiba, Brazil).

DECT has several advantages, it helps avoid an invasive procedure like synovial fluid aspiration, which carries the risk of bleeding, infection, and has low yield in small joints [17]. As demonstrated in Figure 2, gout in small joints like the metatarsophalangeal joints is easily revealed by DECT. Though synovial fluid aspiration is considered the gold standard, it is often deferred in the primary care or emergency care setting where a majority of patients are managed [18]. An accurate diagnosis will reduce inappropriate treatment and institute early interventions to prevent long term complications like joint destruction, and renal and cardiac manifestations.

Another useful characteristic of DECT is the quantification of tophi burden in a joint, which is a terminal manifestation of this disease. This will help monitor the efficacy of urate lowering therapy in addition to diagnosis. As seen in Figure 1, 3D demonstration of DECT illustrates complete resolution of tophi after adequate treatment with febuxostat [4, 15]. This allows quantitative monitoring of tophi and appropriate response to treatment. DECT is also particularly useful to diagnose gout in patients where synovial fluid does not demonstrate any MSU crystals [14]. In a study at Mayo clinic, 30% of patients who had gout diagnosed by DECT, did not have MSU crystals in synovial fluid analysis [14]. This is likely due to MSU crystals clustering around tendons and ligaments instead of the articular space.

Other imaging techniques available for gout are radiography, ultrasonography (USG), conventional CT, and magnetic resonance imaging (MRI). Erosive changes in radiography manifests late in disease, and conventional CT cannot distinguish MSU deposits. On the other hand, USG is an inexpensive and well-accepted diagnostic tool. The double contour sign in USG is characteristic, but there is lack of standardized methodology and sometimes exhaustive scanning is required [19]. These imaging tools therefore lack specificities for implementation on a larger scale [3, 12, 20].

At present, there are five different types of DECT scanners available: Dual source DECT, twin-beam single-source CT with gold filter, rapid kilovoltage-switching source with gemstone scintillator detector, dual-layer multidetector DECT, and dual-scan single source [8].

The amount of radiation involved in DECT ranges between 2 to 3 mSv, though it is dependent on the technology used [12, 21]. This low dose of radiation offers a satisfactory replacement of standard CT scans. Strategies that lead to dose reduction include tube current modulation, iterative reconstruction techniques, and new detector application-specific integrated circuits (ASICs) which integrate photodiode and analog digital converters [21]. DECT also limits radiation to extremities which are not typically radiosensitive regions. This may reduce radiation-related cancer risk, though there is paucity of literature is this area.

Though DECT shows great potential, it is not without limitations. It is user dependent and imaging results should be interpreted with caution by a professional who is well-trained to recognize artifacts. Locations notorious for artefacts were nose, skin, calluses, nail bed, flexor and peroneal tendons, and around arthroplasties [5, 21]. The most common of these is of the nail and nail bed, mainly in the feet, observed in 88% of patients. Figure 1b exhibits the commonly seen nail artifact, which is seen in green pixels [11]. Other artifacts encountered were submillimeter ones caused by noise and beam hardening, seen as an isolated linear pattern. Though common, artifacts are well recognized and easy to rectify for DECT-trained radiologists. False positives can be reduced with measures like physical patient adjustments, increasing gantry speeds for decreasing motion artifacts or adjustment of individual settings during interpretation [11]. 

False positive results are also observed in patients with high grade osteoarthritis. This may be theorized as either a representation of joint damage causing matrix exposure and MSU crystallization or an imaging artifact [14]. Though DECT scan has good accuracy in diagnosing gout in patients with established disease, it appears to have low sensitivity in the first episode of gout in patients with no prior history. This is detailed in a study by Bongartz, et al., where 20% of patients with no prior history of gout failed to demonstrate MSU crystals on DECT imaging. This was attributed to the increasingly small size of deposits in early disease [14]. In a more recent review presented by the US department of Health and Human Services, sensitivity of DECT in patients with initial gout episode was 85% or more, though strength of evidence was low.

## Conclusions

The increase in prevalence of gout in the past two decades may be attributed to lifestyle causing obesity, hyperlipidemia, hypertension, diabetes, and renal disease. This causes a burden on the ambulatory services and was estimated to be 2 million visits for acute attacks annually. Various inflammatory arthritides such as pseudogout, rheumatoid arthritis, psoriatic arthritis, septic arthritis can mimic gout. DECT offers great potential as a screening and diagnostic tool to detect MSU crystals accurately and should be implemented on a larger scale. DECT is a promising tool to diagnose gout reliably and also measure the total uric acid burden in the body.
